# Nystagmus phenotypes in posterior circulation stroke

**DOI:** 10.3389/fneur.2026.1916451

**Published:** 2026-08-25

**Authors:** F. K. Schwarz, P. Rommer, U. Schwarz-Nemec, S. Greisenegger, G. Wiest

**Affiliations:** 1 Department of Neurology, Medical University of Vienna, Vienna, Austria; 2 Comprehensive Center for Clinical Neurosciences and Mental Health, Medical University of Vienna, Vienna, Austria; 3 Department of Biomedical Imaging and Image-guided Therapy, Division of Neuroradiology and Musculoskeletal Radiology, Medical University of Vienna, Vienna, Austria

**Keywords:** acute vestibular syndrome, nystagmus, posterior circulation, stroke, videooculography

## Abstract

**Background:**

Acute vestibular syndrome (AVS) is characterized by sudden, persistent vertigo or dizziness with nausea, vomiting, postural instability, and spontaneous nystagmus. Differentiating peripheral vestibular disorders from posterior circulation stroke remains a major diagnostic challenge in emergency neurology, as vestibulo-cerebellar infarcts may present as “central pseudo-neuritis,” mimicking peripheral vestibular disease. Although the HINTS examination is a valuable bedside tool, its reliability depends on the presence and characterization of nystagmus and is reduced in patients without spontaneous nystagmus.

**Objective:**

To characterize nystagmus phenotypes in posterior circulation stroke using videooculography (VOG) and to identify features that improve recognition of central vestibular pathology in patients with AVS.

**Methods:**

Retrospective single-center descriptive cohort study of 34 patients with posterior circulation stroke confirmed by neuroimaging, who underwent VOG between 2014 and 2026. Eye movements were assessed using VOG and nystagmus features (direction, gaze dependence, fixation suppression, head-shaking responses) were correlated with lesion location.

**Results:**

Thirty-four patients were included; nystagmus was present in 70% (24/35). Of these, 50% showed multiple nystagmus phenotypes and 50% a single pattern. Spontaneous nystagmus (horizontal or vertical) and Head-shaking nystagmus (HSN) both occurred in 62%, while gaze-evoked nystagmus was rare (12%). There was a non-significant trend toward a higher prevalence of HSN in PICA infarctions (OR 4.81) and HSN showed cross-coupled or direction changing features in 80% of patients. No clear correlation was found between the side of the lesion and the direction of nystagmus, nor between fixation and the slow-phase velocity (SPV) of nystagmus.

**Conclusion:**

Nystagmus is a frequent clinical feature of posterior circulation stroke with heterogenous phenotypes. Lesion laterality did not correlate with nystagmus direction, and fixation did not consistently alter SPV. HSN was a frequent sign of central vestibular dysfunction. Performance of the head shaking test may improve detection of central vestibular pathology and complement the HINTS examination in acute vertigo.

## 1 Introduction

The acute vestibular syndrome (AVS) represents a distinct clinical entity defined by the sudden onset of continuous vertigo or dizziness lasting 1 day or more and accompanied by nausea or vomiting, postural instability, and spontaneous nystagmus (1–3). This imbalance may originate from peripheral vestibular structures, most commonly due to acute unilateral vestibulopathy, or from lesions affecting central vestibular pathways, with ischemia in the posterior circulation constituting the most critical differential diagnosis (4, 5).

The HINTS (Head Impulse, Nystagmus, Test of Skew) examination has become an established bedside framework for differentiating peripheral from central causes of AVS (2, 6). Within this examination, careful assessment of nystagmus is of high diagnostic value, as specific nystagmus characteristics provide insight into the anatomical level and pathophysiological mechanism of vestibular dysfunction.

Peripheral vestibular lesions typically produce a unidirectional spontaneous horizontal–torsional nystagmus that follows Alexander’s law, reflecting a static asymmetry in afferent peripheral vestibular activity with preserved function of the central gaze-holding (6, 7). In contrast, central vestibular pathology is usually associated with vertical, pure torsional, direction-changing, or gaze-evoked nystagmus, suggesting dysfunction of central vestibular neural integration, velocity-storage mechanisms, or gaze-holding networks (8–10). However, studies have shown a drop in diagnostic accuracy of HINTS when including acutely dizzy patients without nystagmus (11). Therefore, HINTS cannot be reliably applied in cases without nystagmus (12).

The assessment of head-shaking nystagmus (HSN) may further increase diagnostic sensitivity by challenging central adaptive mechanisms and unmasking latent vestibular asymmetries through activation of the velocity-storage system (8, 13). Central vestibular processing depends on closely coupled interactions between the vestibular nuclei, the nodulus and uvula of the cerebellum, the flocculus/paraflocculus, and the oculomotor integrators of the brainstem, which together convert head velocity signals into stable eye position and gaze commands (9, 13, 14). A large retrospective study has shown that HSN was more frequently observed in a peripheral vestibular disorder group than in the central group. However, the proportion of so-called „perverted “HSN — now more appropriately termed cross-coupled HSN, i.e., when the nystagmus is induced in a plane other than that of head oscillation, was significantly increased in the central group compared to the peripheral vestibular patient group (15). Although a cross-coupled HSN is not pathognomonic for a central vestibular disorder, a recent study shows that its predictive value for a central etiology can be classified as at least moderate (16).

A core mechanism underlying central vestibular nystagmus is dysfunction of the velocity-storage mechanism, a central neural network that extends and spatially aligns vestibular responses beyond the mechanical time of the semicircular canals afferent input. This mechanism is primarily implemented within the vestibular nuclei and is critically modulated by the nodulus and uvula (9, 14). Lesions involving these structures—particularly within the posterior inferior cerebellar artery (PICA) territory—may lead to inappropriate persistence, spatial misalignment, or instability of stored velocity signals. Clinically, this can manifest as spontaneous nystagmus, exaggerated or prolonged HSN, or direction-changing nystagmus while peripheral vestibular function remains intact (10, 13). Furthermore, impaired vestibular integration contributes to the heterogeneity of central nystagmus patterns. Central vestibular processing integrates semicircular canal, otolith, visual, and somatosensory inputs to generate spatially appropriate eye movements. Disruption of this integration—especially at the level of the vestibular nuclei and cerebellum—can result in vertical, torsional, or mixed nystagmus components, abnormal otolith–ocular interactions, and dissociation between vestibulo-ocular reflexes and perceptual verticality (17, 18).

Importantly, central vestibular lesions do not invariably produce overtly “central” nystagmus patterns. Ischemic lesions within the PICA territory may present with nystagmus that closely mimics peripheral vestibular neuropathy, including unidirectional spontaneous nystagmus consistent with Alexander’s law (9, 19). These so-called central pseudo-neuritis presentations are thought to reflect selective involvement of vestibulo-cerebellar structures regulating vestibular tone and velocity storage—such as the nodulus, uvula, and vestibular nuclei—while sparing vertical ocular motor pathways and otolith-mediated skew mechanisms (17, 20).

A failure of gaze holding due to impairment of the neural integrator represents another source of central nystagmus. The neural integrator, involving the horizontal and vertical gaze centers converts eye velocity commands into sustained eye position signals. Damage to these structures results in leaky gaze holding, clinically expressed as gaze-evoked nystagmus with centripetal drift and corrective saccades (14, 21). Cerebellar floccular lesions, in particular, impair adaptive calibration of the neural integrator and may coexist with nystagmus with a peripheral vestibular appearance (22, 23).

In the present study we aim to characterize the spectrum of nystagmus phenotypes in patients with posterior circulation stroke using videooculography (VOG), and to identify features that may facilitate the recognition of central vestibular pathology in patients with AVS.

## 2 Methods

### 2.1 Study population

In this study we retrospectively included all patients (*n* = 34) suffering from an AVS with posterior circulation stroke, documented by neuroimaging, who underwent videooculography (VOG) between 2014 and 2026 at the Department of Neurology, Medical University of Vienna, Austria.

### 2.2 Neuroimaging

Neuroimaging was performed using cerebral magnetic resonance imaging (cMRI) in 33 out of 34 patients. One patient received cranial computer tomography (cCT).

cMRI was performed using standardized imaging protocols. The imaging protocol included diffusion-weighted imaging (DWI) for the detection of acute ischemic lesions, fluid-attenuated inversion recovery (FLAIR) sequences for the assessment of parenchymal lesions and chronic vascular changes, and time-of-flight magnetic resonance angiography (TOF-MRA) for the non-contrast evaluation of intracranial vascular anatomy and the identification of stenotic or occlusive vascular abnormalities. Image acquisition was conducted within the acute or following the acute phase within the standard diagnostic workup.

In one patient, only cCT was performed. The patient developed vertigo following endovascular coiling of a posterior circulation aneurysm, and CT imaging was obtained to exclude intracranial hemorrhage. Additional cMRI was not performed because significant coil-related susceptibility artifacts were anticipated, and the ischemic lesion was already clearly identifiable on CT imaging.

The vascular territories were classified by a neuroradiologist based on the anatomical templates described by Savoiardo et al. (24). Infarct location was determined on diffusion-weighted imaging (DWI) and corresponding apparent diffusion coefficient (ADC) maps, and each lesion was assigned to one of the following arterial territories: posterior inferior cerebellar artery (PICA), superior cerebellar artery (SCA), posterior spinal artery, or basilar artery. Watershed infarcts located at the boundary between two adjacent arterial territories were classified according to the territory that was most involved. In cases of disagreement or ambiguity, a second reader was consulted and consensus was reached.

### 2.3 Nystagmus recording and analysis

VOG and a computer-controlled rotational chair system (VisualEyes™ 515 System, sampling rate 100 fps, Interacoustics A/S, Middelfart, Denmark, Softwareversion 3.2), were used to assess ocular motor function. We recorded eye fixation and spontaneous eye movements, head-shaking nystagmus as well as gaze-evoked nystagmus. SPN was recorded first, with an initial fixation on a light for 10 s, followed by recording of SPN in total darkness for another 20 s. Head-shaking nystagmus was recorded for 20 s after 10 s of active or passive head shaking at a frequency of approximately 3 Hz in total darkness. In cases of co-occurring SPN and HSN, the nystagmus was interpreted as HSN when change of direction or cross-coupled nystagmus occurred, or SPV differed from that of the SPN. Gaze evoked nystagmus was recorded at +/− 22 deg. horizontally and +/− 13 deg. vertically. The analysis of eye movements was performed automatically by system-specific analysis algorithms (Interacoustics) and visually scanned for artifacts by a specialist after recording.

### 2.4 Statistical analysis

Nystagmus phenotypes were examined to investigate potential associations between oculomotor findings and vascular territory. Because individual patients could present with multiple phenotypes, each was analyzed independently as a binary variable (present vs. absent). Head-shaking nystagmus (HSN), gaze-evoked nystagmus (GEN), and spontaneous nystagmus (SPN) were assessed separately, with horizontal and vertical forms of spontaneous nystagmus combined into a single SPN category for analysis.

Given the limited sample sizes within individual vascular territories, infarcts were categorized into posterior inferior cerebellar artery (PICA) and non-PICA groups. Associations between vascular territory and each nystagmus phenotype were evaluated using two-sided Fisher’s exact tests.

Effect sizes were reported as odds ratios (ORs) with corresponding 95% confidence intervals (CIs). In light of the exploratory design and limited sample size, ORs and CIs were included alongside *p*-values to better characterize the magnitude and precision of observed associations.

Categorical variables are presented as counts and percentages. All tests were two-sided, and statistical significance was defined as *p* < 0.05.

## 3 Results

A total of 34 patients with posterior circulation stroke were included in this retrospective descriptive cohort study. The mean age was 70 years with a standard deviation (SD) of 15 years. Sixty-two percent of patients were male (*n* = 21). Imaging was conducted within 24 h (mean) of onset. Twenty-eight patients received VOG within 14 days of symptom onset, 2 within 30 days of onset, 4 patients were referred to VOG at a later date (30–115 days after onset).

### 3.1 Nystagmus phenotypes

Nystagmus was observed in 70% (*n* = 24) of patients. Of these, 50% (*n* = 12) had more than one type of nystagmus, while the other 50% (*n* = 12) had one isolated form of nystagmus. In the overall group of patients with nystagmus, spontaneous nystagmus (SPN) alongside head-shaking nystagmus were the most common findings, occurring both in 62% (*n* = 15) of patients. Of the patients with SPN, 53% (*n* = 8) exhibited horizontal spontaneous nystagmus (hSPN) and 66% (*n* = 10) showed vertical spontaneous nystagmus (vSPN). Gaze-evoked nystagmus (GEN) was found in 12% (*n* = 3) in the overall group of patients with nystagmus.

**Figure 1:**
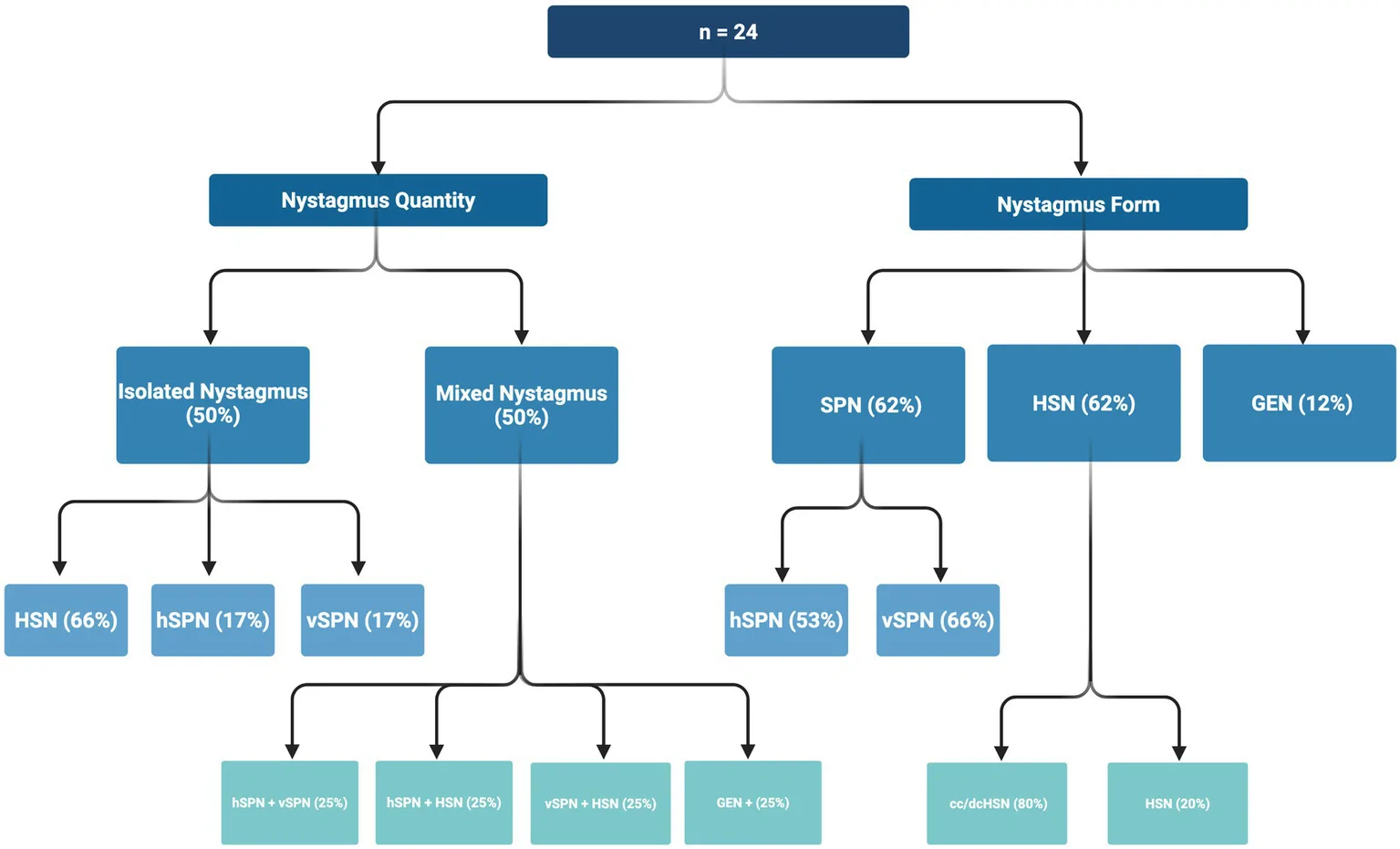
Overview of nystagmus phenotypes and their prevalence in the study population. The figure illustrates the distribution and relative prevalence of the different nystagmus phenotypes identified among the study participants.

In the subgroup of 12 patients with a single isolated form of nystagmus, HSN was the most common, occurring in 66% (*n* = 8) of patients. The remaining patients exhibited SPN, with 17% (*n* = 2) of patients exhibiting a horizontal form and 17% (*n* = 2) of patients exhibiting a vertical form (Figure 1). Of all patients with SPN, hSPN or vSPN (*n* = 15), 33% (*n* = 5) had cross-coupled or direction-changing HSN.

Out of the SPN group, 73% of patients (*n* = 11) showed a decrease in SPV during fixation, whereas the remaining patients (*n* = 4) exhibited an increase.

### 3.2 Direction of nystagmus

Among the 17 patients who had either hSPN and/or hHSN, 41% (*n* = 7) showed ipsilesional nystagmus, while 53% (*n* = 9) showed contralesional nystagmus. Nearly all patients with ipsilesional nystagmus had lesions in the PICA territory (*n* = 6) with the remaining patient showing infarction of the cerebellum in the SCA territory. One patient underwent repeated VOG and showed initial ipsilesional nystagmus, which shifted to contralesional nystagmus at the follow-up examination 6 months later. Concerning contralesional nystagmus, 7 patients had lesions in the PICA territory and 2 in the basilar artery territory.

### 3.3 Vascular territories of the infarction

The affected vascular areas in our stroke patients involved the PICA region in 67% of patients (*n* = 23). 18 patients with PICA infarctions exhibited nystagmus. Lesions in the area of the superior cerebellar artery (SCA) were comparatively rare at 14% (*n* = 5), with three of these patients having watershed infarcts, all of which occurred in the SCA area and additionally involved either the PICA or a section of the basilar artery. Of the patients with infarction of the SCA, four (80%) showed no form of nystagmus, the remaining patient showed an isolated ipsilesional HSN. This patient had suffered from an isolated SCA infarction of the cerebellar hemisphere.

In five additional patients, infarction occurred isolated in the territory of the basilar artery, and in one patient, infarction occurred in the area supplied by the posterior spinal artery.

Patients with infarction in the vascular territory of the basilar artery (*n* = 5) had lesions within the pons (*n* = 3) and mesencephalon (*n* = 2). Two patients exhibited hSPN, both beating to the contralesional side. Two patients had vSPN (Upbeat-Nystagmus (UBN)), one had vHSN (UBN), and one had vGEN. One patient had no nystagmus.

### 3.4 Head shaking nystagmus

Of all 15 patients showing HSN, 66% (*n* = 10) had isolated cerebellar lesions, 13% (*n* = 2) had mixed cerebellar and medullary lesions, 13% (*n* = 2) had isolated lesions in the medulla, and 7% (*n* = 1) had mixed lesions in the pons and pedunculus. In terms of the vascular territories, 86% (*n* = 13) of patients had a lesion within the territory of the PICA, the remaining 2 were lesions in the territory of the basilar artery and the SCA. HSN was purely vertical in 4 patients, purely horizontal in 6 patients and mixed in 5 patients. 80% of patients with HSN (*n* = 12) showed either cross-coupled or direction changing characteristics. The remaining (*n* = 3) consisted of one patient with left beating HSN in combination with a hSPN to the left, and two patients with isolated right beating HSN.

### 3.5 Vertical nystagmus

The most frequent form of vSPN was UBN in 80% of cases (*n* = 8). Downbeat nystagmus (DBN) occurred only (*n* = 2) in cerebellar infarctions, while UBN was associated with isolated medullary infarction (*n* = 4), mixed cerebellar-medullary (*n* = 1) and mesencephalic (*n* = 2) infarctions. The remaining patient with UBN had suffered from an isolated cerebellar infarction. In contrast to the other patients with UBN a significantly higher SPV of 11°/s (mean 5°) was documented. The only other patient with a SPV greater than the mean was the patient with mixed cerebellar-medullary infarction (SPV 7°/s).

### 3.6 Associations of nystagmus and vascular territories

HSN was observed in 13 of 18 patients (72%) with PICA infarction, compared with 2 of 6 patients (33.3%) with non-PICA infarction. Although odds of HSN were fourfold higher in the PICA group, this difference did not reach statistical significance (OR 4.81 (CI 0.51–69.55), Fisher’s exact test *p* = 0.150).

GEN and SPN were similarly distributed across both groups, with no significant association observed between either GEN or SPN and vascular territory (GEN: OR 0.66; SPN: OR 1.0; *p* = 1.00). Overall, none of the individual nystagmus phenotypes demonstrated a statistically significant association with vascular territory. However, HSN exhibited the largest effect size and was more frequently observed in PICA infarctions compared with non-PICA infarctions (Table 1).

**Table 1:** Nystagmus phenotypes in PICA versus non-PICA patients.

Phenotype	PICA (*n* = 19)	Non-PICA (*n* = 6)	Odds ratio	Fisher’s exact *p*
HSN	13/18 (72%)	2/6 (33.3%)	4.81	0.150
GEN	2/18 (11%)	1/6 (16.6%)	0.66	1.00
SPN (combined)	12/18 (66.6%)	4/6 (66.6%)	1	1.00

Distribution and prevalence of head-shaking nystagmus (HSN), gaze-evoked nystagmus (GEN), and spontaneous nystagmus (SPN, combined) in patients with PICA lesions compared to non-PICA controls, with corresponding odds ratios and Fisher’s exact *p* values.

### 3.7 Sensitivity analysis

In a sensitivity analysis limited to patients evaluated during the acute phase (<14 days from symptom onset), the distribution of nystagmus findings closely mirrored that of the full cohort. Among 28 acutely assessed patients, nystagmus was observed in 67.9% (*n* = 19), comparable to 70.1% in the overall population. Within this subgroup, isolated nystagmus patterns were present in 52.6% of patients (*n* = 10), while 47.4% (*n* = 9) exhibited multiple subtypes, indicating a similar balance of isolated and combined patterns as in the full cohort. Head-shaking nystagmus (HSN) and SPN (combined hSPN and vSPN) were observed with equal frequency (63.2%, *n* = 12). GEN was infrequent (10.5%, *n* = 2), consistent with its low prevalence in the overall cohort (12%). Overall, these findings indicate that the nystagmus patterns observed in our study are representative, as a restriction to the acute phase (<14 days) yielded a comparable distribution.

## 4 Discussion

Our data demonstrate that nystagmus was a key feature in over 70% of cases of posterior circulation strokes. Isolated and combined forms of nystagmus were equally balanced in our study, as were horizontal and vertical components in the described spontaneous nystagmus. In terms of the territorial supply areas, it was predominantly infarcts in the PICA area that caused the nystagmus described.

The findings highlight the prominent role of HSN in this cohort. HSN was not only frequently observed overall, but also represented the most common isolated nystagmus pattern, suggesting that it can occur independently of other vestibular and oculomotor abnormalities.

In the cohort of 15 patients presenting with HSN, cerebellar involvement was the most common neuroanatomical correlate. The majority of lesions (86%) occurred within the territory of the PICA, which supplies the inferior cerebellum and portions of the lateral medulla. The predominance of PICA-territory lesions suggests that structures supplied by this vascular region, including the vestibulocerebellum and adjacent brainstem pathways, may play an important role in the mechanisms underlying HSN. These finding are also well supported by the current literature (8).

Head-shaking nystagmus (HSN) may represent a valuable addition to the HINTS examination, particularly for differentiating acute unilateral vestibulopathy (AUVP) from posterior inferior cerebellar artery (PICA) infarction. In our cohort 80% of patients with HSN (*n* = 12) exhibited either cross-coupled or direction-changing HSN, while only three patients showed unidirectional responses. Cross-coupled and direction-changing HSN, is generally regarded as a sign of central vestibular pathology. This phenomenon is thought to arise from disruption of the velocity-storage mechanism within the vestibulocerebellum, particularly structures such as the nodulus and uvula (8, 25).

A large retrospective study has shown that HSN was more frequently observed in a peripheral vestibular disorder group than in the central group. However, the proportion of cross-coupled HSN, i.e., when the nystagmus is induced in a plane other than that of head oscillation, or direction-changing HSN was significantly increased in the central group compared to the peripheral vestibular patient group (15). Although a cross-coupled HSN is not pathognomonic for a central vestibular disorder, a recent study shows that its isolated predictive value for a central etiology can be classified as at least moderate (16). In cases of vestibular neuritis cross-coupled HSN has not been described (16). In our study HSN was more frequently observed in PICA infarctions (OR 4.81), but this estimate is exploratory given the wide confidence interval and association was not statistically significant (*p* = 0.150), limiting its interpretive value in the context of so-called “pseudo-neuritis.” Taken together, these findings emphasize the potential diagnostic value of HSN, even in the absence of additional forms of nystagmus. HSN may provide an additional bedside indicator of central pathology and should therefore possibly be integrated into the HINTS examination, contributing to a more reliable differentiation between peripheral vestibular and central vestibular causes of acute vertigo in the absence of SPN (26).

Concerning vSPN, DBN notably occurred exclusively in patients with cerebellar infarctions, whereas UBN was associated with a broader range of lesion locations. The exclusive association of DBN with cerebellar infarction is consistent with the established role of the vestibulocerebellum, particularly the flocculus and paraflocculus, in maintaining vertical gaze stability and modulating vestibulo-ocular reflex pathways (8).

Analysis of the SPV of the nystagmus revealed additional noteworthy features. One patient with isolated cerebellar infarction and UBN demonstrated a markedly elevated SPV of 11°/s, which was substantially higher than the cohort mean of 5°/s. The only other patient with an SPV exceeding the mean was the individual with mixed cerebellar–medullary infarction, who showed an SPV of 7°/s.

These findings suggest that lesions involving cerebellar structures may produce more pronounced vertical vestibular imbalance, resulting in higher nystagmus intensity. UBN due to interruption of cerebellar pathways from the perihypoglossal nuclei to the flocculus are discussed (27) and increase in SPV in lesions affecting these pathways have been proposed (28). From a pathophysiological perspective the cerebellum normally plays a critical role in damping and calibrating vestibular signals; therefore, damage to these regulatory circuits could lead to stronger spontaneous nystagmus (13) and an increase in SPV during fixation. In contrast, gaze-evoked nystagmus was underrepresented in our study (6, 22).

Concerning the change of SPV during fixation in our patient group, SPV was decreased in 73% (*n* = 11) of patients with SPN, which is in contrast to conventional teachings, but has been reported before (29, 30). A reduction in SPV during visual fixation is, however, consistent with preserved fixation suppression, which depends on the smooth pursuit system. The smooth pursuit system is primarily mediated by the cerebellar flocculus/paraflocculus complex and the nodulus (29). In this group, most infarctions were located in the brainstem (*n* = 8). Brainstem infarctions that spare the cerebellar peduncles are likely to preserve these cerebellar structures functionally, thereby maintaining fixation suppression; in such cases, the nystagmus may reflect vestibular nuclear involvement rather than dysfunction of cerebellar modulatory circuits (31).

Among the patients who exhibited an increase in SPV during fixation (*n* = 4), indicating impaired fixation suppression, 3 had cerebellar lesions. This observation is in line with prior evidence identifying the nodulus and floccular regions as key structures whose damage is associated with failure of fixation suppression (29).

Concerning nystagmus directionality relative to lesion side, our findings are consistent with prior studies describing both ipsi- and contralesional nystagmus in central vestibular lesions, confirming that these do not invariably produce overtly “central” nystagmus patterns. Lesions affecting structures such as the nodulus and uvula can reduce inhibitory input to the vestibular nuclei, resulting in relative contralateral hyperactivity and contralesional nystagmus. Ipsilesional nystagmus occurred exclusively with lesions of the PICA territory, suggesting acute dysfunction of vestibular nuclei or adjacent cerebellar structures (19). One patient demonstrated a change from ipsilesional to contralesional nystagmus over 6 months, also supporting the concept of dynamic central vestibular compensation (32).

Out of five patients with infarction of the SCA territory only one patient showed ipsilesional HSN. These findings are consistent with current literature regarding frequency and direction of nystagmus in SCA infarctions (33).

There are limitations to this study. First, for methodological reasons, we were unable to record purely torsional eye movements, meaning that this aspect of central vestibular nystagmus could not be assessed. In addition, only 28 patients could be examined using VOG during the acute phase of posterior circulation stroke, as soon as they were physically able to do so. Seven patients could only be examined at a later stage, so that incipient compensation mechanisms cannot be ruled out here. Similarly, due to the study design, the assessment of positional nystagmus was not included in the analysis of this cohort. Finally, the monocentric and retrospective nature of the study further limits the generalizability of the findings.

## 5 Conclusion

The head-impulse test is usually used at the bedside to rule out a peripheral vestibular deficit in AVS. In addition, the skew deviation test in HINTS is used to detect a lesion of the central otolithic pathway. However, there are few pathognomonic signs for the diagnosis of central vestibular nystagmus. Our data suggest that the inclusion of the HSN may serve as an additional helpful tool for raising suspicion of a posterior circulation stroke at the bedside.

## Data Availability

The original contributions presented in the study are included in the article/supplementary material, further inquiries can be directed to the corresponding author.

## References

[1] Kim JS Newman-Toker DE Kerber KA Jahn K Bertholon P Waterston J . Vascular vertigo and dizziness: diagnostic criteria. J Vestib Res. (2022) 32:205–22. doi: 10.3233/VES-210169, 35367974 PMC9249306

[2] Kattah JC Talkad AV Wang DZ Hsieh YH Newman-Toker DE. HINTS to diagnose stroke in the acute vestibular syndrome: three-step bedside oculomotor examination more sensitive than early MRI diffusion-weighted imaging. Stroke. (2009) 40:3504–10. doi: 10.1161/STROKEAHA.109.551234, 19762709 PMC4593511

[3] CobanFA TarnutzerAA. The epidemiology and clinical presentation of acute vestibular syndromes: a systematic review of the literature. J Neurol. (2026) 273:173. doi: 10.1007/s00415-026-13720-541770418

[4] Hotson JR Baloh RW. Acute vestibular syndrome. N Engl J Med. (1998) 339:680–5. doi: 10.1056/NEJM199809033391007, 9725927

[5] Edlow JA Selim MH. Atypical presentations of acute cerebrovascular syndromes. Lancet Neurol. (2011) 10:550–60. doi: 10.1016/S1474-4422(11)70069-2, 21601162

[6] Nham B Akdal G Young AS Özçelik P Tanrıverdizade T Ala RT . Capturing nystagmus in the emergency room: posterior circulation stroke versus acute vestibular neuritis. J Neurol. (2023) 270:632–41. doi: 10.1007/s00415-022-11202-y, 35849153 PMC9886594

[7] Fracica E Hale D Gold DR. Diagnosing and localizing the acute vestibular syndrome - beyond the HINTS exam. J Neurol Sci. (2022) 442:120451. doi: 10.1016/j.jns.2022.120451, 36270149

[8] Huh YE Kim JS. Patterns of spontaneous and head-shaking nystagmus in cerebellar infarction: imaging correlations. Brain J Neurol. (2011) 134:3662–71. doi: 10.1093/brain/awr269, 22036958

[9] Lee H Sohn SI Cho YW Lee SR Ahn BH Park BR . Cerebellar infarction presenting isolated vertigo: frequency and vascular topographical patterns. Neurology. (2006) 67:1178–83. doi: 10.1212/01.wnl.0000238500.02302.b4, 17030749

[10] Choi KD Kim JS. Head-shaking nystagmus in central vestibulopathies. Ann N Y Acad Sci. (2009) 1164:338–43. doi: 10.1111/j.1749-6632.2008.03737.x, 19645923

[11] Kerber KA Meurer WJ Brown DL Burke JF Hofer TP Tsodikov A . Stroke risk stratification in acute dizziness presentations: a prospective imaging-based study. Neurology. (2015) 85:1869–78. doi: 10.1212/WNL.0000000000002141, 26511453 PMC4662702

[12] Tarnutzer AA Koohi N Lee SU Kaski D. Diagnostic errors in the acutely dizzy patient-lessons learned. Brain Sci. (2025) 15:55. doi: 10.3390/brainsci15010055, 39851423 PMC11764146

[13] Leigh RJ Zee DS. The Neurology of Eye Movements. New York, NY: Oxford University Press (2015).

[14] Beh SC Frohman TC Frohman EM. Cerebellar control of eye movements. J Neuroophthalmol. (2017) 37:87–98. doi: 10.1097/WNO.0000000000000456, 27643747

[15] Yang TH Lee JH Oh SY Kang JJ Kim JS Dieterich M. Clinical implications of head-shaking nystagmus in central and peripheral vestibular disorders: is perverted head-shaking nystagmus specific for central vestibular pathology? Eur J Neurol. (2020) 27:1296–303. doi: 10.1111/ene.14161, 31999861

[16] Lee SU Tarnutzer AA. Usefulness of nystagmus patterns in distinguishing peripheral from central acute vestibular syndromes at the bedside: a critical review. J Clin Neurol. (2025) 21:161–72. doi: 10.3988/jcn.2025.0105, 40308011 PMC12056143

[17] Zwergal A Strupp M Brandt T Büttner-Ennever JA. Parallel ascending vestibular pathways: anatomical localization and functional specialization. Ann N Y Acad Sci. (2009) 1164:51–9. doi: 10.1111/j.1749-6632.2009.04461.x, 19645880

[18] Dieterich M Brandt T. Ocular torsion and tilt of subjective visual vertical are sensitive brainstem signs. Ann Neurol. (1993) 33:292–9. doi: 10.1002/ana.410330311, 8498813

[19] Kim HA Yi HA Lee H. Recent advances in cerebellar ischemic stroke syndromes causing Vertigo and hearing loss. Cerebellum. (2016) 15:781–8. doi: 10.1007/s12311-015-0745-x, 26573627

[20] Kim HA Lee H. Isolated vestibular nucleus infarction mimicking acute peripheral vestibulopathy. Stroke. (2010) 41:1558–60. doi: 10.1161/STROKEAHA.110.582783, 20489171

[21] Tarnutzer AA Weber KP Schuknecht B Straumann D Marti S Bertolini G. Gaze holding deficits discriminate early from late onset cerebellar degeneration. J Neurol. (2015) 262:1837–49. doi: 10.1007/s00415-015-7773-9, 25980905

[22] Baier B Dieterich M. Incidence and anatomy of gaze-evoked nystagmus in patients with cerebellar lesions. Neurology. (2011) 76:361–5. doi: 10.1212/WNL.0b013e318208f4c3, 21263137

[23] Strupp M Kremmyda O Adamczyk C Böttcher N Muth C Yip CW . Central ocular motor disorders, including gaze palsy and nystagmus. J Neurol. (2014) 261:542–58. doi: 10.1007/s00415-014-7385-9, 25145891 PMC4141156

[24] Savoiardo M Bracchi M Passerini A Visciani A. The vascular territories in the cerebellum and brainstem: CT and MR study. AJNR Am J Neuroradiol. (1987) 8:199–209. 3105277 PMC8335382

[25] Lee SU Choi JY Kim HJ Park JJ Zee DS Kim JS. Impaired tilt suppression of post-rotatory nystagmus and cross-coupled head-shaking nystagmus in cerebellar lesions: image mapping study. Cerebellum. (2017) 16:95–102. doi: 10.1007/s12311-016-0772-2, 26969184

[26] Kim JS Ahn KW Moon SY Choi KD Park SH Koo JW. Isolated perverted head-shaking nystagmus in focal cerebellar infarction. Neurology. (2005) 64:575–6. doi: 10.1212/01.WNL.0000150729.87682.79, 15699406

[27] Meling TR Nouri A May A Guinand N Vargas MI Destrieux C. Upbeat vertical nystagmus after brain stem cavernoma resection: a rare case of nucleus intercalatus/nucleus of roller injury. J Neurol. (2020) 267:2865–70. doi: 10.1007/s00415-020-09891-4, 32458196 PMC7501124

[28] Tian J Otero-Millan J Zee DS Kheradmand A. Upbeat nystagmus with an unusual velocity-decreasing and increasing waveform: a sign of gaze-holding dysfunction in the Paramedian tracts in the medulla? Cerebellum. (2023) 22:148–54. doi: 10.1007/s12311-022-01376-6, 35133635

[29] Kim HA Yi HA Lee H. Failure of fixation suppression of spontaneous nystagmus in cerebellar infarction: frequency, pattern, and a possible structure. Cerebellum. (2016) 15:182–9. doi: 10.1007/s12311-015-0688-2, 26082303

[30] Mantokoudis G Wyss T Zamaro E Korda A Wagner F Sauter TC . Stroke prediction based on the spontaneous nystagmus suppression test in dizzy patients: a diagnostic accuracy study. Neurology. (2021) 97:e42–51. doi: 10.1212/WNL.0000000000012176, 33986142 PMC8312858

[31] Lee SH Kim JM Schuknecht B Tarnutzer AA. Vestibular and ocular motor properties in lateral medullary stroke critically depend on the level of the medullary lesion. Front Neurol. (2020) 11:390. doi: 10.3389/fneur.2020.00390, 32655466 PMC7325917

[32] Dutia MB. Mechanisms of vestibular compensation: recent advances. Curr Opin Otolaryngol Head Neck Surg. (2010) 18:420–4. doi: 10.1097/MOO.0b013e32833de71f, 20693901

[33] Lee H Kim HA. Nystagmus in SCA territory cerebellar infarction: pattern and a possible mechanism. J Neurol Neurosurg Psychiatry. (2013) 84:446–51. doi: 10.1136/jnnp-2012-303177, 23172866

